# Effects of Feeding a Mycotoxin Binder on Nutrient Digestibility, Alkaloid Recovery in Feces, and Performance of Lambs Fed Diets Contaminated with Cereal Ergot

**DOI:** 10.3390/toxins10080312

**Published:** 2018-08-01

**Authors:** Kim Stanford, Mary Lou Swift, Yuxi Wang, Tim A. McAllister, John McKinnon, Barry Blakley, Alex V. Chaves

**Affiliations:** 1 Livestock Research Section, Alberta Agriculture and Forestry, Lethbridge, AB T1J 4V6, Canada; 2 Department of Agriculture, Food and Nutritional Sciences, University of Alberta, Edmonton, AB T6G 2P5, Canada; MaryLou.Swift@trouwnutrition.com; 3 Agriculture and Agri-Food Canada, Lethbridge Research and Development Centre, Lethbridge, AB T1J 4B1, Canada; yuxi.wang@agr.gc.ca (Y.W.); tim.mcallister@agr.gc.ca (T.A.M.); 4 Department of Animal and Poultry Science, University of Saskatchewan, Saskatoon, SK S7N 5B4, Canada; john.mckinnon@usask.ca; 5 Department of Veterinary Biomedical Sciences, University of Saskatchewan, Saskatoon, SK S7N 5A8, Canada; brb237@mail.usask.ca; 6 School of Life and Environmental Sciences, University of Sydney, Sydney 2006, NSW, Australia; alex.chaves@sydney.edu.au

**Keywords:** cereal ergot, mycotoxin binder, lamb performance, nutrient digestibility

## Abstract

As contamination with cereal ergot has been increasing in western Canada, this study evaluated impacts of feeding a mycotoxin binder (Biomin^®^ II; BB) on nutrient digestibility, alkaloid recovery in feces, and lamb growth performance. Forty-eight ram lambs (25.9 ± 1.4 kg) were randomly assigned to one of four barley-based diets: Control (C), no added alkaloids, Control + BB fed at 30 g/head per day (CBB); Ergot, 2564 ppb total *R* + *S* epimers (E); Ergot + BB, 2534 ppb *R + S* epimers (EBB). Lambs were fed ab libitum for up to 11 weeks until slaughter at >46 kg live weight. Both average daily gain (ADG) and gain/feed ratio were greater (*p* < 0.01) for lambs fed C and CBB diets as compared with those containing added ergot, although dry matter intake was not affected by dietary ergot or BB. Serum prolactin concentrations were two times higher in EBB- compared with E-fed lambs (*p* < 0.05), although both were lower than in C or CBB (*p* < 0.001) lambs. Rectal temperatures were greater in lambs receiving dietary ergot (*p* ≤ 0.001) than in C- and CBB-fed lambs. In a digestibility study using eight ram lambs, treatment with BB increased neutral detergent fiber (NDF) digestibility (*p* = 0.01). Nitrogen retention (g) was greater (*p* < 0.05) for lambs receiving C or CBB compared with ergot-contaminated diets. Feces of EBB lambs had 38.5% greater (*p* < 0.001) recovery of alkaloids compared with those fed E. Based on sparing of prolactin, BB may reduce impacts of ergot alkaloids by increasing their excretion in feces. Accordingly, concentrations of dietary alkaloids, which would not harm sheep, would be increased by feeding BB.

## 1. Introduction

Over the past 15 years, contamination of cereal grains with ergot has markedly increased in western Canada [1]. Consequently, livestock have been increasingly exposed to a number of alkaloids produced by *Claviceps purpurea* fungi, including ergocornine, ergocristine, ergocryptine, ergotamine, ergometrine, ergosine, and corresponding *S* epimers [2]. Common symptoms of exposure of livestock to ergot alkaloids are diverse and may include a reduction in feed intake and/or reduced growth performance [3], decreased serum prolactin [4], agalactia [5], vasoconstriction leading to impaired thermoregulation [6], gangrenous extremities [4], reduced fetal growth [7], and abortion [8]. Multiple alkaloids are generally present simultaneously in livestock feeds, although individual ergot bodies may not contain all alkaloids [9]. Toxic effects of alkaloids may be additive [10], synergistic [11], or less than additive [12]. As a further complication, alkaloids may interconvert between the more-biologically active *R* and the less-active *S* epimers [13].

Strategies to reduce impacts of mycotoxins, such as ergot alkaloids on livestock, range from preventing fungal infections through crop rotations and selecting crop varieties that resist infection [14] to applying a variety of physical or chemical treatments to the grain after it is contaminated. Grain cleaning techniques have largely eliminated ergotism as a human disease [6], but this process concentrates ergot in the grain screenings that are used as livestock feed. Binders that adsorb mycotoxins to their surface and prevent their absorption in the digestive tract are another harm-reduction strategy [15], but they may also adversely affect absorption of vitamins, minerals, or antibiotics [16]. Binders are most commonly clays or yeast-derivatives [17] and may also contain enzymatic components for biotransformation and detoxification of mycotoxins [18], as well as growth promoters or immune enhancers [15].

Binding capacity varies with the physical and chemical nature of the adsorbent (total charge, charge distribution, pore size) and the characteristics of the mycotoxin (polarity, solubility, shape, and charge distribution) [19]. Adsorption agents have been useful for preventing aflatoxicosis, but results for other mycotoxins have been more variable [20]. To our knowledge, in vivo use of binders for ergot alkaloids has not been studied, although several in vitro studies have shown promise [21,22]. As in vivo studies evaluating the ability of binders to reduce negative impacts of ergot alkaloids are limited, the objective of the present study was to evaluate growth performance, nutrient digestion and recovery of ergot alkaloids in feces of lambs fed pelleted diets containing a mycotoxin binder.

## 2. Results

### 2.1. Alkaloid Concentrations and Profiles

Concentration of total and individual alkaloids did not differ between Control (no added alkaloids, C) and Control + Biomin^®^ II (BB) (fed at 30 g/head/day; CBB) or between Ergot (2564 ppb total alkaloids; E) and Ergot + BB (2533 ppb total alkaloids; EBB) diets (Table 1). Accordingly, adding the alkaloid binder to diets did not influence the analysis of *R* or *S* epimers in the diets.

### 2.2. Nutrient Digestion by Lambs

Although digestibility of dry matter (DM), organic matter (OM), crude protein (CP), and acid detergent fiber (ADF) was not affected by dietary ergot or BB, digestibility of neutral detergent fiber (NDF) was higher (*p* = 0.01) in lambs fed BB compared with those fed diets without BB (Table 2). The BB did not negatively impact nutrient digestibility or N metabolism, as other than higher NDF digestibility, effects of BB were not significant (*p* > 0.17). Intake of N and N output did not differ among diets, but nitrogen retention (g) increased (*p* < 0.05) in lambs receiving control diets (C, CBB) as compared with those receiving diets containing added ergot (E, EBB). Similarly, N retained as a percentage of intake and percentage of N retained of that digested averaged 4.9% greater (*p* < 0.05) in lambs receiving diets without added ergot. Concentrations of alkaloids in feces of lambs fed C or CBB were below detectable limits (Table 3). For diets with added ergot, BB increased (*p* < 0.001) recovery of *R* epimers in feces from an average of 2.6% of that consumed in lambs receiving E to 3.6% for lambs fed EBB. Effects of BB were consistent for all individual alkaloids, with each showing greater recovery (*p* < 0.001) in feces from lambs fed EBB.

### 2.3. Lamb Performance, Serum Prolactin, Rectal Temperature, and Carcass Characteristics 

Initial live weight (LW), final LW, and dry matter intake (DMI) were not affected by dietary ergot or by BB (Table 4), although both average daily gain (ADG) and feed conversion (gain/feed) were greater (*p* < 0.05) in lambs receiving C or CBB as compared with those receiving diets containing added ergot. Adding BB to diets increased serum prolactin concentrations of lambs (*p =* 0.05), with that of lambs fed EBB two times higher than that of lambs fed E. However, prolactin concentrations of EBB lambs remained lower (*p* < 0.001) than those of C- and CBB-fed lambs. Average rectal temperature was highest in lambs fed diets with added ergot (*p* < 0.05), but the difference between those and C and CBB lambs were small (0.26 °C). Adding ergot to diets or adding BB did not affect hot carcass weight, dressing percentage or grade rule (GR) measurement.

## 3. Discussion

### 3.1. Alkaloid Concentrations and Profiles

The recommended dosage of BB ranges from 15 to 30 g/head per day, depending on concentrations of mycotoxins in the feed [23]. As alkaloid concentrations in the diets with added ergot approached the allowable limits for Canadian sheep [24], a dosage of 30 g/head per day was used. If BB had interfered with measurement of alkaloids in diets, the results of the current study would have been less clear. Fortunately, concentrations of individual epimers and total alkaloids were similar for dietary treatments with or without BB, likely because the alkaloid extraction protocol freed the bound alkaloids. Total alkaloid concentrations of diets with added ergot were similar to the highest concentration used in a previous study [12]. However, even though the same source of ergot-contaminated screenings was used in both studies, concentrations of individual alkaloids differed, a reflection of the variability in concentrations of alkaloids in individual ergot bodies [9]. Compared with the previous study, the most marked changes in alkaloid concentrations included ergotamine (+43%), ergocristine (+35%), ergocryptine (−31%), and ergometrine (−20%). Consequently, while total alkaloid concentrations were similar across the two studies, changes in alkaloid profiles likely contributed to differences in animal responses between the present and our previous study [12]. 

### 3.2. Nutrient Digestibility

Both NDF and ADF digestibility were linearly reduced with increasing ergot alkaloid concentration in our previous study, where lambs were fed up to 432 ppb *R* epimers [25]. However, no changes in nutrient digestibility were noted by Coufal-Majewski et al. [12] using the same source and concentration of contaminated screenings as this study. Accordingly, as digestibility of NDF was impacted in the present study only by BB and not by dietary ergot, ergot alkaloids appear to inconsistently influence fiber digestibility. Differences among lambs, variability in alkaloid profiles and concentrations between studies, or a combination of these factors is likely responsible for the observed inter-study variation. Alkaloids present in tall fescue seed have been shown to inhibit fiber digestion in sheep [26], although ergovaline, the primary alkaloid present in tall fescue, is not prevalent in cereal ergot [27].

Impacts of mycotoxin binders on nutrient digestibility may be affected by the types of mycotoxins present. Dänicke et al. [28] suggested that BB depressed fiber digestion in lambs exposed to *Fusarium*-contaminated wheat. In contrast, we found that BB improved NDF digestibility in lambs fed CBB or EBB diets. Currently, BB contains proprietary plant extracts designed to compensate for adverse conditions in the gastrointestinal tract created by mycotoxins [29] in addition to bentonites and diatomaceous earth, which can adsorb polar mycotoxins [30]. As a result of the multiple constituents of BB, it was not possible to determine which ingredients were responsible for improving NDF digestibility. The BB did not negatively impact nutrient digestibility or N metabolism. Accordingly, BB meets a primary requirement for a mycotoxin binder in that it did not impair nutrient absorption [19].

A lower N retention in lambs fed diets containing added ergot was not noted in our previous lamb study [12], although reduced digestibility of protein has been reported in pigs receiving diets containing 4.7 ppm total ergot alkaloids [*R* + *S* epimers] [3]. Impacts of alkaloids on N metabolism in other studies using pigs have varied depending on the concentrations [31] and types of alkaloids [3]. Regardless of overall impacts of ergot alkaloids on N metabolism, BB did not improve N retention, which was lower in lambs fed diets with added ergot as compared with those receiving C or CBB. In contrast, Kiyothong et al. [30] found that BB improved digestibility of crude protein and NDF in dairy cattle fed diets contaminated with a mixture of *Fusarium* mycotoxins. Many *Fusarium* mycotoxins contain a highly-reactive epoxide group that is directly involved in their toxicity [15], while ergot alkaloids do not contain a comparable functional group. As both expoxidase and binding components of BB would target *Fusarium* mycotoxins, it is not surprising that effectiveness of BB is influenced by the type of mycotoxin present. 

Mycotoxin binders have been developed primarily to adsorb mycotoxins and prevent their absorption in the gastro-intestinal tract [14], and we are unaware of previous in vivo studies that have examined their ability to bind ergot alkaloids. In the present study, BB increased excretion of *R* epimers in feces by an average of 38.5%, although excretion varied by alkaloid and ranged from 0.6 to 7.0% of the individual alkaloids consumed. Alkaloid recovery in feces was lower than that reported in our previous study [12], but was similar to the 5% recovery of fescue alkaloids in sheep feces reported by others [32].

### 3.3. Animal Performance, Rectal Temperature, and Serum Prolactin

In the present study, lambs fed diets containing added ergot had reduced ADG as compared with those fed C or CBB, with similar reductions in ADG noted for lambs receiving 2447 ppb total alkaloids [12]. Negative responses to dietary ergot in the present study also included reduced feed conversion efficiency, a result similar to that reported for diets containing 432 ppb *R* epimers [25]. However, BB did not improve the ADG or gain/feed in EBB lambs.

Feed intake by lambs in this and our previous studies has been consistently unaffected by cereal ergot, an observation that contrasts to studies with chickens [33] and pigs [3], but is similar to those with calves [34]. Feed intake has been improved, along with performance measures in some studies of BB in dairy cattle fed diets contaminated with *Fusarium* [30], but not in dairy cattle receiving diets contaminated with aflatoxins [29]. Similar to the present study, BB neither increased feed intake nor performance measures in a study of pigs receiving diets contaminated with *Fusarium* mycotoxins [35]. The BB has received EU registration based on its capacity to bind aflatoxin [23], but its utility may vary with the type of mycotoxin, although similarities in the binding of aflatoxin and ergopeptine alkaloids in vitro have been reported [36]. The BB was chosen for evaluation in the present study because of its binding constituents, including kaolin clay, as previous in vitro studies had demonstrated binding of ergot alkaloids to a variety of clays [21,22,36]. In contrast to the ergot alkaloid binders previously evaluated in vitro, BB was available in Canada and licensed for use in Canadian feeds, enabling slaughter of the lambs through conventional channels.

Lambs receiving diets contaminated with fescue alkaloids have previously been shown to have increased rectal temperatures and reduced serum prolactin concentrations [37], similar to E and EBB lambs in this study. Increased rectal temperatures after exposure to ergot are thought to be related to alkaloid-induced vasoconstriction interfering with the capacity for thermoregulation [4,6,37]. Although increased rectal temperatures in lambs fed ergot were small, lambs were housed in a ventilated barn and thermal stress to lambs may have been increased if they had been kept in outdoor pens, as would be typical of commercial lamb production. Increased body temperatures and other symptoms of inflammation have been linked to immune responses of pigs exposed to *Fusarium* mycotoxins [38]. Accordingly, differences in the immune status of animals and existing extent of inflammation prior to consumption of contaminated feed may possibly explain the variation in animal responses to ergot alkaloids and other mycotoxins [39]. Lambs in the present study were healthy, which may explain their relatively limited responses to high concentrations of alkaloids. 

In our previous studies with lambs, serum prolactin concentrations were a better predictor of the impact of ergot on growth than were dietary concentration of alkaloids. Prolactin concentration has already been identified as a suitable indicator for exposure to fescue alkaloids [4]. That EBB lambs had increased serum prolactin concentrations demonstrates that BB reduced the impact of alkaloids through increased fecal excretion. The sparing effect of BB on prolactin is important as it confirms that the mycotoxin binder directly reduced the negative physiological impacts of the alkaloids as opposed to acting as a non-specific growth promoter.

Concentrations of alkaloids in E and EBB were high, approaching the maximum allowable in diets for Canadian sheep [24]. As BB did not improve growth performance or feed conversion efficiency, the costs for feeder lambs would outweigh benefits. Perhaps greater concentrations of BB would improve N retention and growth performance, but greater concentrations may also lead to the binding of nutrients [19]. Based on the sparing effect on prolactin and improved NDF digestibility noted in EBB as compared with E-fed lambs, BB may have more utility for breeding animals receiving high-forage diets. Conditions such as agalactia are directly related to inhibition of prolactin secretion in animals exposed to ergot alkaloids [40], but the ability of BB to prevent this economically-devastating condition, or otherwise protect breeding ruminants from negative impacts of ergot alkaloids requires further investigation. As BB increased excretion of alkaloids in feces, adding BB to diets for sheep would increase the concentration of alkaloids, which could be fed without causing harm, although breakpoints would have to be established for feeder lambs as compared with breeding stock, which may also be affected by the alkaloid profile of the contaminated feed. 

## 4. Materials and Methods

All experiments were conducted between June and September 2017 at the Lethbridge Research Centre (LRC) of Agriculture and Agri-Food Canada. Protocol #1507 was reviewed and approved on 29 October 2016 by the LRC Animal Care Committee according to the guidelines of the Canadian Council on Animal Care [41]. 

### 4.1. Ergot Source, Alkaloid Determination 

Ergot-contaminated barley screenings were sourced from a previous study [12] in which similar concentrations of screenings were included in the diet. To prepare diets, screenings were ground through a 1 mm screen using a Wiley^®^ Cutting Mill (Thomas Scientific, Swedesboro, NJ, USA). Ground screenings were substituted for barley and four different pelleted diets were made each in a single batch (2500 kg; Table 5). The mycotoxin binder (Biomin^®^ II, Biomin Canada Inc., Mont-St.-Hillaire, QC, Canada) contained diatomaceous earth, kaolin clay, yeast and plant extracts, and enzymes targeted for the degradation of zearalenones and trichothecenes. Diets were Control (C; no added alkaloids); Control + BB fed at 30 g/head per day; CBB); Ergot (2564 ppb total *R* + *S* epimers; E); and Ergot (2534 ppb total *R* + *S* epimers + BB fed at 30 g/head per day; EBB; Table 1). The BB was added to the appropriate diets prior to pelleting. During the course of the study, a 500 g sample of each diet was collected and analyzed four times for thirteen ergot alkaloids (i.e., chanoclavine, ergocornine, ergocorninine, ergocristine, ergocristinine, ergocryptine, ergocryptinine, ergometrine, ergometrinine, ergosine, ergosinine, ergotamine, and ergotaminine) by Biomin Research Center (Tulln, Austria) using HPLC-MS/MS [42]. A subsample (5.0 g) of the diet was ground through a 1-mm screen and stored at room temperature overnight in 20 mL of extraction solvent (acetonitrile/water/acetic acid 79:20:1, *v*/*v*/*v*) in the dark. Samples were then extracted for 90 min and centrifuged for 2 min at 1500× *g.* The pellet was discarded and 350 µL aliquots of the supernatant was then diluted with solvent (acetonitrile/water/acetic acid 79:20:1, *v*/*v*/*v*) and mixed. A total of 5 µL of extracted alkaloids was then injected into an Agilent 1290 HPLC (Aligent Technologies Inc., Waldbronn, Germany) combined with an Applied Biosystems 5500 QTrap mass spectrometer. Chromatographic separation was performed at 25 °C on a Gemini^®^ C_18-_column, 150 × 4.6 mm internal diameter, 5 µm particle size equipped with a C_18_ security guard cartridge, 4 × 3 mm internal diameter (Pheomenex, Torrance, CA, USA).

### 4.2. Nutrient Digestion and Metabolism of Lambs

Eight newly-weaned Canadian Arcott × Rideau Arcott ram lambs that had not been previously exposed to dietary ergot were selected based on similar initial LW (20.5 ± 0.49 kg), to estimate the digestibility of the diets using a cross-over design. Each of the four periods lasted 21 day, with 14 day for adaption and 7 day of sample collection. Lambs were fed once daily, and based on the previous 4 day of intake were restricted to 95% of ad libitum intake during the week of total collection. Health of lambs was visually monitored daily. Lambs were shorn and pre-fitted with strap-on canvas fecal collection bags 7 day prior to the first collection. During collections, lambs were individually fed in crates with transparent panels, which allowed visual contact with the other lambs and minimized stress. Feces and urine were collected daily in the last 4 day of each period and analyzed for DM, OM, CP, NDF, and ADF as described by Coufal-Majewski et al. [25]. Samples of each diet (500 g) were collected during each of the four periods and analyzed for the same constituents, as were feces and urine. Dried ground subsamples (500 g) of feces collected in each period from each diet were also analyzed for ergot alkaloid *R* epimers.

### 4.3. Performance Study

Forty-eight newly-weaned ram lambs were blocked by weight and randomly assigned to one of the four diets (Table 5). Lambs were individually fed and received both feed and water ad libitum, with orts and feed consumed determined daily. Lambs were weighed weekly with rectal temperatures measured at weighing using a digital thermometer (GLA M750, GLA Agricultural Electronics, San Luis Obispo, CA, USA). Ambient temperature in the barn was also recorded three times daily during the study. Blood samples were collected by venipuncture from 36 lambs at 35 kg body weight (BW), and at slaughter weight (≥46 kg). Lambs chosen for prolactin analyses received experimental diets for at least three weeks prior to the initial blood collection, with an interval of at least three weeks between bleedings. Larger lambs, which were >30 kg at initiation of the study, were excluded from prolactin analyses. Serum was obtained by centrifugation (2000× *g* for 15 min at 4 °C) and stored in 1 mL screw-cap tubes in duplicate at –80 °C. Prolactin concentrations were then determined in serum using a double antibody radioimmunoassay as described by Coufal-Majewski et al. [25]. 

Lambs remained on feed over a period of 6 to 11 weeks until reaching slaughter weight. Thirty-five lambs were shipped to a commercial abattoir for slaughter (Sungold Meats, Innisfail, AB, Canada) with hot carcass weight, dressing percentage, and grade rule (GR) measurement determined according to standards of the Canadian Food Inspection Agency [43]. Lamb health and well-being was monitored several times daily and one lamb was removed from the study as a result of urinary calculi.

### 4.4. Statistical Analyses 

Digestibility and nitrogen metabolism estimates were analyzed by two-way analysis of variance (ANOVA) using the MIXED procedure of SAS (SAS Inst. Inc., Cary, NC, USA) in a crossover design with diet (control vs. ergot) and treatment (control vs. BB) as fixed effects and lamb within diet × treatment as a random effect. For the performance study, initial live weight, final live weight, ADG, feed efficiency, serum prolactin, and carcass data were analyzed as a completely randomized design with diet (control vs. ergot) and treatment (control vs. BB) as a fixed effects and lamb within diet × treatment as a random effect. Average daily gain was determined by dividing weight gain (initial LW–final LW) by the number of days in the study. Feed efficiency was calculated as the ratio between ADG and DMI (g of live weight gain/g DMI). Prolactin data were normalized by log transformation prior to analyses. A repeated measures analysis was used for DMI analyses, with week as the repeated variable and lamb within diet × treatment a random effect. Treatment means were compared using the least squares mean (LSMEANS) linear hypothesis test of SAS (SAS Inst. Inc., Cary, NC, USA). Significance for all analyses was declared at *p* ≤ 0.05.

## Figures and Tables

**Table 1:** Mean concentrations of alkaloid *R* and *S* epimers (ppb) in experimental diets fed to lambs. C—Control; CBB—Control + BB; E—Ergot; EBB—Ergot + BB.

Alkaloid	C		CBB ^1^		E		EBB	
	*R* Epimer	*S* Epimer	*R* Epimer	*S* Epimer	*R* Epimer	*S* Epimer	*R* Epimer	*S* Epimer
Chanoclavin	0		0		1		1	
Ergocornine/inine	1	0	10	2	106	79	104	77
Ergocristine/inine	4	1	6	2	915	246	985	260
Ergotcryptine/inine	0	0	0	0	159	70	140	65
Ergometrine/inine	0	1	3	1	321	3	292	4
Ergosine/inine	0	0	0	0	149	47	151	41
Ergotamine/inine	1	0	10	1	419	50	369	44
Total epimers	6	2	29	6	2070	494	2042	491
Total alkaloids	8		36		2564		2533	

^1^ Biomin^®^ II (BB), containing diatomaceous earth, kaolin, plant and yeast extracts, esterase, and epoxidase, fed at 30 g/head per day.

**Table 2:** Nutrient digestion and nitrogen metabolism in lambs (*n* = 8) comparing diets with or without added ergot and the presence or absence of a mycotoxin binder (treatment).

	Diet ^1^		Treatment ^1^			Probability			
Digestibility %	C, CBB	E, EBB	C, E	CBB, EBB	SEM ^2^	Diet	Treat	Period	Diet × Treat
Dry matter	72.6	72.0	72.1	72.4	0.5	0.43	0.65	0.19	0.94
Organic matter	74.8	72.0	72.1	72.4	0.5	0.40	0.18	0.16	0.92
Crude protein	74.8	74.1	73.9	75.0	0.6	0.89	0.88	0.01	0.66
Neutral detergent fiber	46.3	46.0	44.2	48.1	0.8	0.79	0.01	0.25	0.01
Acid detergent fiber	27.3	29.0	27.6	28.7	1.1	0.30	0.49	0.27	0.83
N metabolism									
N intake, g	45.8	45.7	45.9	45.7	1.1	0.97	0.91	<0.001	0.58
N digested, g	32.7	32.8	32.8	32.8	0.8	0.93	0.98	<0.001	0.53
N retained, g	11.2	9.4	10.3	10.3	0.5	0.02	0.93	0.02	0.32
N output, g	35.6	36.6	36.4	35.8	1.1	0.52	0.71	<0.001	0.84
% of N retained (intake)	25.3	21.2	23.3	23.1	1.1	0.02	0.90	<0.001	0.50
% of N retained (digested)	35.4	29.7	32.6	32.4	1.4	0.01	0.95	<0.001	0.54

^1^ C, no added alkaloids; CBB no added alkaloids + mycotoxin binder fed at 30 g/head per day; E, 2564 ppb total alkaloids (*R* + *S* epimers); EBB, 2533 ppb total alkaloids + mycotoxin binder. ^2^ SEM, standard error of the mean

**Table 3:** Recovery (%) of individual *R* epimers in feces of lambs (*n* = 8) fed diets with or without added ergot in the presence or absence of a mycotoxin binder.

	Diet ^1^					Probability	
Alkaloid	C ^2^	CBB ^2^	E	EBB	SEM	Diet	Period
Ergocornine	0 ^a^	0 ^a^	2.3 ^b^	3.1 ^c^	0.1	<0.001	0.85
Ergocristine	0 ^a^	0 ^a^	0.8 ^b^	1.0 ^c^	0.05	<0.001	0.60
Ergocryptine	0 ^a^	0 ^a^	4.5 ^b^	7.0 ^c^	0.3	<0.001	0.58
Ergometrine	0 ^a^	0 ^a^	0.6 ^b^	0.8 ^c^	0.04	<0.001	0.93
Ergosine	0 ^a^	0 ^a^	2.5 ^b^	3.1 ^c^	0.07	<0.001	0.23
Ergotamine	0 ^a^	0 ^a^	4.9 ^b^	6.6 ^c^	0.1	<0.001	0.31

^a,b,c^ Means in a row with different superscripts differ (*p* < 0.001). ^1^ C, no added alkaloids; CBB no added alkaloids + mycotoxin binder fed at 30 g/head per day; E, 2564 ppb total alkaloids (*R* + *S* epimers); EBB, 2533 ppb total alkaloids + mycotoxin binder. ^2^ For both C and CBB diets, alkaloids in feces below limit of detection.

**Table 4:** Growth performance, rectal temperature (°C), and carcass characteristics of lambs compared diets with or without added ergot and the presence or absence of a mycotoxin binder (treatment). Lambs received experimental diets for 6 to 11 weeks.

	Diet ^1^		Treatment ^1^			Probability		
Parameter	C, CBB	E, EBB	C, E	CBB, EBB	SEM	Diet	Treat	Diet × Treat
Growth (*n* = 47)								
Initial body weight (kg)	25.9	25.8	25.9	25.8	1.4	0.98	0.98	0.79
Final body weight (kg)	48.1	48.0	48.5	47.6	0.6	0.89	0.64	0.71
Dry matter intake (g/day)	1470.6	1430.7	1454.2	1446.7	43.3	0.35	0.86	0.18
Average daily gain (g/day)	413.8	349.8	379.4	384.2	13.7	0.01	0.81	0.27
Feed conversion (gain:feed)	0.28	0.24	0.26	0.26	0.01	0.003	0.77	0.84
LN **^2^** serum prolactin (µg/L)	5.33	2.18	3.48	4.03	0.14	<0.001	0.05	0.32
Rectal temperature °C	39.12	39.38	39.28	39.22	0.07	<0.001	0.41	0.34
Carcass characteristics (*n* = 35)								
Hot carcass weight (kg)	21.1	20.9	21.1	20.8	0.3	0.64	0.54	0.73
Dressing percentage	43.2	42.8	43.1	42.9	0.7	0.57	0.78	0.34
Grade rule (mm)	16.2	15.0	15.5	15.8	0.6	0.17	0.71	0.20

^1^ C, no added alkaloids; CBB no added alkaloids + mycotoxin binder fed at 30 g/head per day; E, 2564 ppb total alkaloids (*R* + *S* epimers); EBB, 2533 ppb total alkaloids + mycotoxin binder. ^2^ LN, normal log.

**Table 5:** Diet composition.

Ingredients, g/kg	C	CBB	E	EBB
Barley grain	540.0	520.0	537.5	517.5
Alfalfa pellets (18% CP)	270.0	270.0	270.0	270.0
Canola meal	168.0	168.0	168.0	168.0
Limestone	3.0	3.0	3.0	3.0
Sheep Mineral ^1^	10.0	10.0	10.0	10.0
Vitamin A, D, E ^2^	0.25	0.25	0.25	0.25
Deccox ^3^	0.132	0.132	0.132	0.132
Ergot screening	0.0	0.0	2.52	2.52
Canola oil	10.0	10.0	10.0	10.0
Biomin^®^ II ^4^	0.0	20.0	0.0	20.0
**Dry matter (DM) and chemical composition (g/kg, Analytical DM basis)**				
Organic matter	925.5 (±1.3)	919.1 (±0.3)	926.3 (±1.5)	917.5 (±3.0)
Crude protein (CP)	190.5 (±14.5)	194.8 (±8.4)	191.3 (±43.5)	193.7 (±32.5)
Neutral detergent fiber	264.7 (±104.6)	256.7 (±88.4)	257.9 (±32.0)	280.2 (±30.2)
Acid detergent fiber	121.2 (±11.1)	124.0 (±12.0)	127.3 (±23.2)	130.4 (±24.0)

^1^ Sheep mineral constitutes (%): salt 92.6, potassium magnesium sulfate 4.979, zinc sulfate 0.921, magnesium sulfate 0.835, organic iodine 0.014, 1% selenium premix 0.143, cobalt carbonate 0.004, canola oil 0.398. ^2^ Vitamin ADE constitutes: vitamin A 10,000,000 IU, vitamin D 1,000,000 IU, vitamin E 10,000 IU/kg. ^3^ Deccox active ingredient: decoquinate (6%; Zoetis, Kirkland, QC, Canada). ^4^ Biomin^®^ II (BB) includes kaolin clay, yeast extracts, diatomaceous earth, plant extracts, esterase, and epoxidase. No other clay-based pellet binder included in diets.
